# Aerobic training enhances muscle deoxygenation in early post-myocardial infarction

**DOI:** 10.1007/s00421-016-3326-x

**Published:** 2016-01-12

**Authors:** Shun Takagi, Norio Murase, Ryotaro Kime, Masatsugu Niwayama, Takuya Osada, Toshihito Katsumura

**Affiliations:** Faculty of Sport Sciences, Waseda University, 2-579-15 Mikajima, Tokorozawa, Saitama 359-1192 Japan; Department of Sports Medicine for Health Promotion, Tokyo Medical University, 6-1-1 Shinjuku, Shinjuku-ku, Tokyo, 160-8402 Japan; Department of Electrical and Electronic Engineering, Faculty of Engineering, Shizuoka University, 3-5-1 Johoku, Naka-ku, Hamamatsu, Shizuoka 432-8561 Japan

**Keywords:** Cycling training, Ischemic heart disease, Microcirculation, Near-infrared spectroscopy, Oxygen transport

## Abstract

**Purpose:**

Exercise-induced skeletal muscle deoxygenation is startling by its absence in early post-myocardial infarction (MI) patients. Exercise training early post-MI is associated with reduced cardiovascular risk and increased aerobic capacity. We therefore investigated whether aerobic training could enhance the muscle deoxygenation in early post-MI patients.

**Methods:**

21 ± 8 days after the first MI patients (*n* = 16) were divided into 12-week aerobic training (TR, *n* = 10) or non-training (CON, *n* = 6) groups. Before and after intervention, patients performed ramp bicycle exercise until exhaustion. Muscle deoxygenation was measured at vastus lateralis by near-infrared spectroscopy during exercise.

**Results:**

Aerobic training significantly increased peak oxygen uptake (VO_2_) (18.1 ± 3.0 vs. 22.9 ± 2.8 mL/kg/min), decreased the change in muscle oxygen saturation from rest to submaximal and peak exercise (∆SmO_2_; 2.4 ± 5.7 vs. −7.0 ± 3.4 %), and increased the relative change in deoxygenated hemoglobin/myoglobin concentration from rest to submaximal (−1.5 ± 2.3 vs. 3.0 ± 3.6 μmol/L) and peak exercise (1.1 ± 4.5 vs. 8.2 ± 3.5 μmol/L). Change in total hemoglobin/myoglobin concentration in muscle was not significantly affected by training. In CON, no significant alterations were found after 12 weeks in either muscle deoxygenation or peak VO_2_ (18.6 ± 3.8 vs. 18.9 ± 4.6 mL/kg/min). An increase in peak VO_2_ was significantly negatively correlated with change in ∆SmO_2_ (*r* = −0.65) and positively associated with change in ∆deoxy-Hb/Mb at peak exercise (*r* = 0.64) in TR.

**Conclusions:**

In early post-MI patients, aerobic training enhanced skeletal muscle deoxygenation, and the enhancement was related to increased aerobic capacity.

## Introduction

Exercise training after onset of heart attack is important for heart disease patients to improve aerobic capacity. In chronic heart failure (CHF) patients, an improvement of aerobic capacity by exercise training can be explained not only by increased cardiac output in the heart (Dubach et al. 1997), but also increased peak muscle blood flow (Sullivan et al. 1988), increased arterial venous oxygen (O_2_) difference (Dubach et al. 1997), and increased mitochondrial density and oxidative enzyme activity in skeletal muscle (Belardinelli et al. 1995; Minotti et al. 1990; Sullivan et al. 1988). However, the ability for patients to increase peripheral variables associated with aerobic capacity by exercise training soon after myocardial infarction (MI) has not been well established. Previous studies partly demonstrated that an aerobic training intervention improved peripheral factors such as a cross-sectional area of thigh muscle (Murabayashi et al. 1997) and muscle oxidative capacity (Cottin et al. 1996) in early post-MI patients. However, these studies had no control (i.e. non-training) group. It is possible that cardiovascular responses and aerobic capacity may be gradually improved over the natural time course in early after onset of MI patients (Wohl et al. 1977). Therefore, peripheral factors and their relationship to the improvement of aerobic capacity may be altered without exercise training. To strictly test the effect of training in early post heart disease patients, the effect of the natural time course should be also investigated.

The balance between O_2_ delivery and utilization in exercising muscle can be measured noninvasively by near-infrared spectroscopy (NIRS) technique during dynamic exercise (Hamaoka et al. 2007), and the technique has been widely used in the research areas of sports science and exercise physiology. Recently, Takagi et al. (2014) reported that in early post-MI patients, the absence of muscle deoxygenation measured by NIRS technique was observed during submaximal and peak exercise, and the deoxygenation abnormalities were related to reduced systemic aerobic capacity. To date, it is unclear whether aerobic exercise training improves skeletal muscle deoxygenation abnormalities in early post-MI patients. In addition, changes in muscle deoxygenation through exercise training might be related to improvement of peak aerobic capacity, if exercise training alters muscle deoxygenation. An increase in aerobic capacity reduces cardiovascular-associated morbidity and mortality, as well as an increase in physical activity (Myers et al. 2004). Therefore, an increase in peak aerobic capacity via exercise training is important even for early post-MI patients. Moreover, early initiation of moderate intensity aerobic training may be more beneficial to improve peak aerobic capacity than late initiation (Johnson et al. 2014). Assessing the effects of aerobic training on muscle deoxygenation can be useful and helpful in understanding the increase in peripheral variables and its relation to increased peak aerobic capacity in MI patients. The aim of this study was to investigate the effects of aerobic exercise training on muscle deoxygenation and its relationship to aerobic capacity.

## Methods

### Patients

Inclusion criteria of the subjects were: (1) fewer than 6 weeks after onset of their first heart attack, (2) age between 40 and 75 years old, and (3) ability to perform exercise testing. All participants had received a percutaneous coronary stent as part of their post-MI clinical treatment. Initially, 23 MI patients were recruited between June 2010 and March 2012 from the cardiac rehabilitation center at Tokyo Medical University Hospital. Patients with peripheral vascular disease, post-infarction angina, critical arrhythmia, (unstable) heart failure, or lung disease were excluded. As a result, 16 early post-MI patients (21 ± 8 days after the first MI) were chosen to participate and invited to engage in exercise training. However, 6 of the 16 subjects were unable to participate in exercise training due to their work schedules. The authors decided to exclude them from the training group, and consequently, the 16 MI subjects were divided into an aerobic exercise training (TR, *n* = 10) group and a non-training (CON, *n* = 6) group (Table 1). Mean left ventricular ejection fraction (LVEF) was determined by echocardiography. Peak creatine kinase was measured during the course of their disease. Hemoglobin concentration was measured before exercise testing by blood sampling from the antecubital vein. All medication dosages taken by the MI patients remained stable during the study. Before this study, all patients participated in approximately five sessions of exercise training as part of cardiac rehabilitation in the hospital. A session consisted of cycling exercise for 30 min at the individual’s three metabolic equivalents. The study protocol was approved by the Medical Research Ethics Committee of Tokyo Medical University (#1405) and was conducted in accordance with the Declaration of Helsinki. All subjects were informed of the purpose of the study, and written informed consent was obtained.

**Table 1:** Physical and clinical characteristics and medication profiles

	TR (*n* = 10)		CON (*n* = 6)	
	Before	After	Before	After
Age (years)	59 ± 10	NA	61 ± 9	NA
Height (cm)	164.9 ± 9.6	NA	165.5 ± 7.2	NA
Weight (kg)	68.5 ± 13.9	68.8 ± 13.7	65.9 ± 12.2	66.1 ± 12.0
Men/women	8/2	–	5/1	–
Hemoglobin (g dL^−1^)	13.1 ± 1.8	13.9 ± 1.5	13.5 ± 1.6	13.8 ± 1.1
Number of days after onset of MI	20 ± 7	NA	23 ± 8	NA
LVEF (%)	56 ± 6	55 ± 4	55 ± 9	55 ± 9
Fat layer thickness at VL (mm)	4.86 ± 1.52	4.90 ± 1.39	4.57 ± 0.60	4.55 ± 0.57
Peak creatine kinase (IU L^−1^)	3674 ± 1472	NA	3084 ± 2544	NA
Site of MI (%)				
Anterior	60	NA	67	NA
Inferior	40	NA	33	NA
Number of diseased vessels (%)				
1	60	NA	67	NA
2	20	NA	17	NA
3	20	NA	17	NA
Medication (%)				
ACE inhibitor	70	–	57	–
Angiotensin II receptor antagonist	20	–	14	–
Beta-blockers	80	–	71	-
Calcium channel antagonist	30	–	29	
Diuretics	30	–	29	
Diabetes (%)	10	–	14	–
Dyslipidemia (%)	70	–	71	–
Hypertension (%)	10	–	14	–

*TR* training group, *CON* non-training group, *LVEF* left ventricular ejection fraction, *VL* vastus lateralis, *MI* myocardial infarction, *ACE* angiotensin converting enzyme, *NA* not available, – no change between before and after intervention

### Exercise training

Training frequency was set to two sessions per week for 12 weeks. Each session mainly consisted of cycling exercise for 30 min at the individual’s estimated lactate threshold (LT) minus 10 W. Before and after the main cycling exercise, subjects performed stretching for 10 min and cycling at a half of LT minus 10 W for 1 min, as a warm up and cool down. The intensity of cycling training was increased gradually (5 W) over the training period if the heart rate (HR) of estimated LT level attained during baseline exercise testing was not attained during training. All training was monitored by physicians. In CON, none of the subjects were involved in any type of exercise training over the 12 weeks.

### Exercise testing (aerobic capacity and cardiorespiratory variables measurement)

In both groups, exercise testing was administered before and after 12 weeks to evaluate the effects of training. After a 3-min warm up at 10 W, the subjects performed 10 W min^−1^ ramp cycling exercise until exhaustion (Strength Ergo 8, Fukuda-Denshi, Japan). During exercise, pedal frequency of 50 rpm was maintained by keeping time with a metronome, as used in previous studies (Takagi et al. 2014). Subjects were supervised by the investigators, and verbal encouragement was given when needed. All subjects exercised until they could no longer sustain the required cadence. The HR was monitored continuously during the exercise by a 12-lead electrocardiogram (ML-9000 Stress Test System; Fukuda-Denshi, Japan), and pulmonary O_2_ uptake (VO_2_) and carbon dioxide production (VCO_2_) were assessed breath-by-breath (AE310S; Minato Medical Science, Japan) to determine peak VO_2_ and estimated LT, using the V-slope method and substantiated in the profiles of the end-tidal partial pressures of O_2_ and CO_2_, the ventilatory equivalents for VO_2_ and VCO_2_, and respiratory exchange ratio (RER) (Beaver et al. 1986; Takagi et al. 2014; Wasserman et al. 1973). Immediately after maximal exercise, all subjects cycled at 10 W for 3 min. The seat and handlebar height remained constant for each subject in exercise testing before and after training.

### VO_2_ response

The ∆VO_2_/∆WR slope during ramp exercise was calculated by the linear regression of VO_2_ with work rate, from 4 min after the beginning of the ramp loading up to the point that represented 80 % of the total duration of ramp exercise (Boone et al. 2008; Day et al. 2003). Ideally, the sub-LT region of the response should have been analyzed, however, since the fit was based on only a very small amount of data, it would have become unstable in the ∆VO_2_/∆WR slope. The mean response time (MRT) in VO_2_ during ramp exercise was calculated as the time from onset of the ramp workload to the point of intersection between the baseline VO_2_ during warm up and a backward extrapolation of the VO_2_ vs time slope (Boone et al. 2008; Takagi et al. 2014). The baseline VO_2_ was defined as the mean of VO_2_ during the last 2 min of warm up. For some subjects, workloads 4 min after onset of ramp loading were higher than workloads at estimated LT. Therefore, the whole slope of ∆VO_2_/∆WR and the MRT were calculated using data from six MI patients in TR and four MI patients in CON.

### Skeletal muscle deoxygenation measurements

Relative changes from rest in oxygenated hemoglobin/myoglobin concentration (∆oxy-Hb/Mb), deoxygenated hemoglobin/myoglobin concentration (∆deoxy-Hb/Mb) and total hemoglobin/myoglobin concentration (∆total-Hb/Mb), and muscle O_2_ saturation (SmO_2_) were continuously assessed at the belly of the vastus lateralis (VL) muscle in the left leg by near-infrared spatial resolved spectroscopy (NIR_SRS_). The measurement site in VL was defined as 30 % of the length between the patella and the greater trochanter, above the patella (Takagi et al. 2013, 2014). The measured signals were defined as the values averaged over the last 10 s at rest, every 20 W, and peak exercise. During the recovery time of re-saturation, the half-recovery time of SmO_2_ (T1/2reoxy) was determined as the time for 50 % reoxygenation of SmO_2_ from the exhaustion level to the peak level (Ichimura et al. 2006). During rest periods without pedaling, subjects sat on the bicycle with the left foot on the pedal, at the lowest position.

In this study, a two-wavelength (770 and 830 nm) light-emitting diode NIR_SRS_ (Astem Co, Japan) was employed. The probe consisted of one light source and two photodiode detectors, and the optode distances were 20 and 30 mm, as were used in previous studies for measuring muscle deoxygenation (Kime et al. 2010; Takagi et al. 2014). The data sampling rate was 1 Hz. Although a previous study demonstrated that fat layer thickness influences NIRS data due to light scattering, the effects of fat layer thickness can be corrected in relative changes in Hb/Mb (Niwayama et al. 2000) or SmO_2_ (Niwayama et al. 2007). The specifications of correction for the influence of fat layer thickness have been fully described (Niwayama et al. 2000, 2007). In this study, fat layer thickness at the measurement site of the VL muscles was measured individually before and after intervention, using an ultrasound device (LogiQ3, GE-Yokokawa Medical Systems, Japan). Subsequently, we utilized an NIRS device which had built-in fat-correction software (Astem Co, Japan) and calculated relative changes in Hb/Mb and SmO_2_ with correction for the influence of fat layer thickness, based on individual value of fat layer thickness before and after intervention. Even though an upper limit of fat layer thickness was designated as 10 mm to correct for the light-scattering effects in this study, fat layer thickness at the measurement site was within ~10 mm in all subjects before and after intervention (Table 1).

### Statistical analysis

All data are given as mean ± standard deviation. Differences in NIRS, power output, cardiorespiratory variables at peak exercise and estimated LT, VO_2_ response, T1/2reoxy, and demographics variables were analyzed by 2-way repeated-measures analysis of variance (ANOVA) with group and intervention (before–after) as factors. To compare changes in variables during submaximal exercise between before and after intervention over power output and percent of peak VO_2_, 2-way ANOVA was used, with intervention (before–after) and power output and percent of peak VO_2_ as factors. Because one subject could not exercise at more than 62 W before training and 65 W after training, repeated measures between groups were limited to rest, 20, 40, and 60 W compared as a function of power output. As a function of percent of peak VO_2_, differences between groups were determined at rest, 20, 40, 60, and 80 % of peak VO_2_. Similarly, 2-way ANOVA was also adapted to test the baseline condition between groups. Where appropriate, the Bonferroni post hoc test was performed to determine specific significant differences. Pearson’s correlation coefficient was employed to determine the relationship between variables. The ANOVA and correlation coefficient were analyzed by SPSS version 17.0 J (SPSS Inc., Chicago, IL). For all statistical analyses, significance was accepted at *p* < 0.05.

## Results

### Exercise training in TR

Although training frequency was set at two exercise sessions/week for 12 weeks, the subjects exercised 20 ± 3 sessions during the 12-week training period, as their schedules permitted. Initial training intensity (i.e. estimated LT minus 10 W) determined by exercise testing before training was 42 ± 11 W, and the final training intensity was 51 ± 9 W.

### Physical and clinical variables (Table 1)

There were no significant interactions or significant main effects in any physical or clinical variables between before and after the intervention.

### Cardiorespiratory variables

**Fig. 1:**
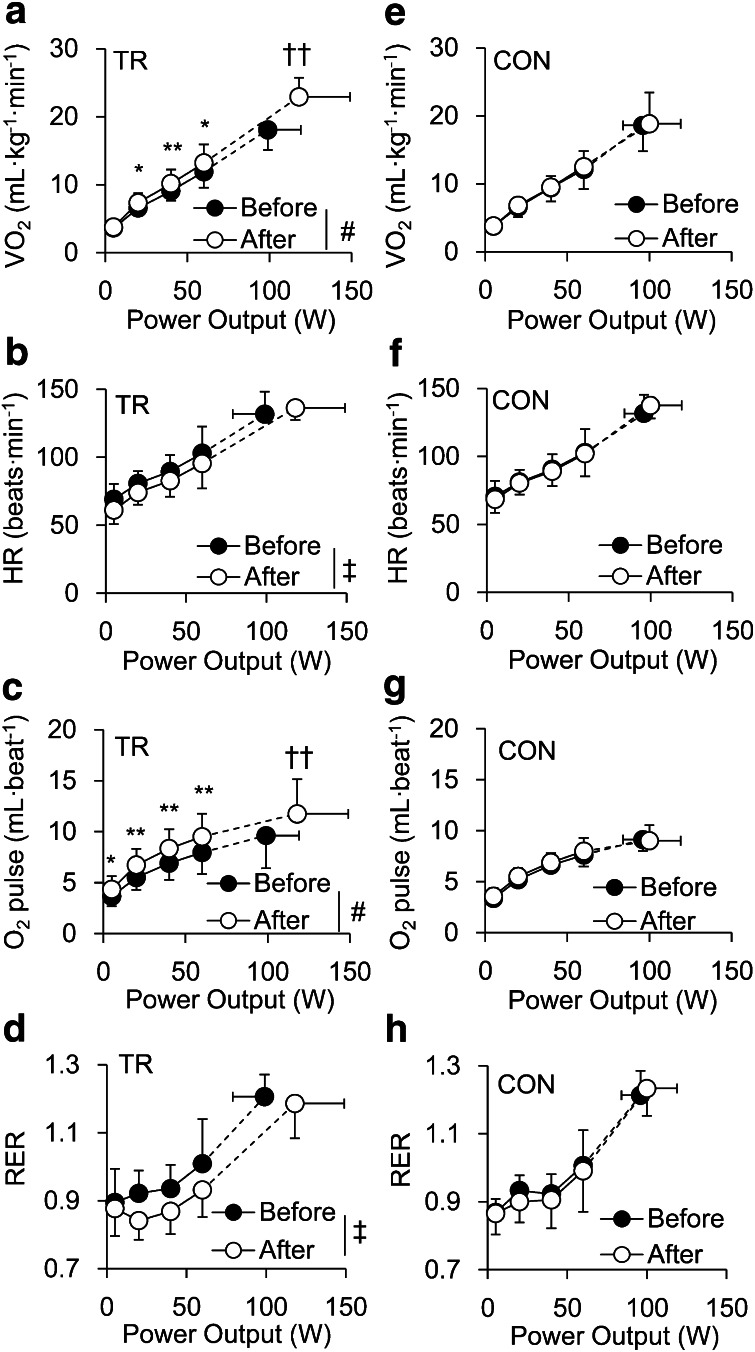
Cardiorespiratory responses in TR and CON. Change in oxygen uptake (VO_2_: **a**), heart rate (HR: **b**), oxygen pulse (**c**), and respiratory exchange ratio (RER: **d**) during ramp cycling exercise expressed as a function of power output before (*closed circles*) and after (*open circles*) intervention in exercise training (TR) and non-training groups (CON). Significant difference between before and after intervention during submaximal exercise (*p < 0.05, **p < 0.01). Significant difference between before and after intervention at peak exercise (^†^ *p* < 0.05, ^††^ *p* < 0.01). Significant intervention (before–after) × power output interaction (^#^ *p* < 0.05). Significant main effects of TR (^‡^ *p* < 0.05). For the sake of clarity, symbols indicating a significant difference between power output before and after intervention have been omitted

During submaximal exercise, in TR, there were significant intervention (before–after) × power output interactions for changes in VO_2_ (*p* < 0.05, Fig. 1a) and O_2_ pulse (*p* < 0.05, Fig. 1c). Even though no significant interactions were observed, a significant main effect of intervention was observed in HR (*p* < 0.05, Fig. 1b) and RER (*p* < 0.05, Fig. 1d). VO_2_ was significantly higher after training at 20–60 W, and O_2_ pulse was significantly greater after training at rest and 20–60 W. When expressed as a function of peak VO_2_ (i.e. percent of peak VO_2_), there were significant interactions for changes in VO_2_ (*p* < 0.01) and O_2_ pulse (*p* < 0.01) in TR. Significantly higher VO_2_ and O_2_ pulse were also observed during submaximal exercise after intervention in TR. In contrast, there were no significant interactions or significant main effects in HR and RER during submaximal exercise. In CON, both expressed as a function of power output and a function of peak VO_2_, no significant changes were found after intervention in any cardiorespiratory variables. Moreover, at baseline, no significant difference was observed between groups in any cardiorespiratory variables during submaximal exercise.

At peak exercise, there were significant group × intervention (before–after) interactions for VO_2_ (*p* < 0.01), O_2_ pulse (*p* < 0.05), and power output (*p* < 0.05). In TR, peak VO_2_ (*p* < 0.01), O_2_ pulse at peak exercise (*p* < 0.01), and peak power output (*p* < 0.01) were significantly increased by training compared to before training (Fig. 1a, c; Table 2), and peak VO_2_ was significantly higher in TR than CON after training (*p* < 0.05, Table 2). In contrast, there were no significant interactions or main effects in HR and RER at peak exercise. At estimated LT, there were significant group × intervention (before–after) interactions for VO_2_ (*p* < 0.05) and power output (*p* < 0.05). In TR, both VO_2_ and power output were significantly increased after training (*p* < 0.01) at estimated LT (Table 2).

**Table 2:** Aerobic capacity and VO_2_ response during ramp exercise

	TR (*n* = 10)		CON (*n* = 6)	
	Before	After	Before	After
Peak				
VO_2_ (mL/kg/min)	18.1 ± 3.0	22.9 ± 2.8^**,#^	18.6 ± 3.8	18.9 ± 4.6
Power Output (W)	99 ± 20	119 ± 32**	96 ± 12	100 ± 19
Heart rate (bpm)	132 ± 16	136 ± 9	132 ± 14	138 ± 10
Estimated LT				
VO_2_ (mL/kg/min)	11.6 ± 1.6	14.4 ± 2.4**	11.9 ± 4.0	12.1 ± 3.6
Power Output (W)	52 ± 11	63 ± 16**	52 ± 11	53 ± 11
VO_2_ response				
∆VO_2_/∆WR slope	10.3 ± 0.4	10.3 ± 1.0	10.8 ± 2.0	10.6 ± 0.6
Mean response time in VO_2_ (s)	83 ± 14	61 ± 13**	80 ± 27	79 ± 20
T1/2reoxy (s)	31 ± 5	31 ± 5	32 ± 12	30 ± 10

Significant difference between before and after intervention (* *p* < 0.05, ** *p* < 0.01)

Significant difference between TR and CON (^#^ *p* < 0.05)

*TR* training group, *CON* non-training group, *VO*_*2*_ oxygen uptake, *LT* lactate threshold, *WR* work rate, *T1/2reoxy* half-recovery time of muscle oxygen saturation

There was a significant group × intervention (before–after) interaction for MRT (*p* < 0.05) but not for ∆VO_2_/∆WR slope (*p* = 0.80). The MRT was significantly lower after training than before in TR (*p* < 0.01), and the MRT tended to be lower in TR than CON after training (*p* = 0.09, Table 2). No significant main effects were found in ∆VO_2_/∆WR slope.

### Muscle deoxygenation

**Fig. 2:**
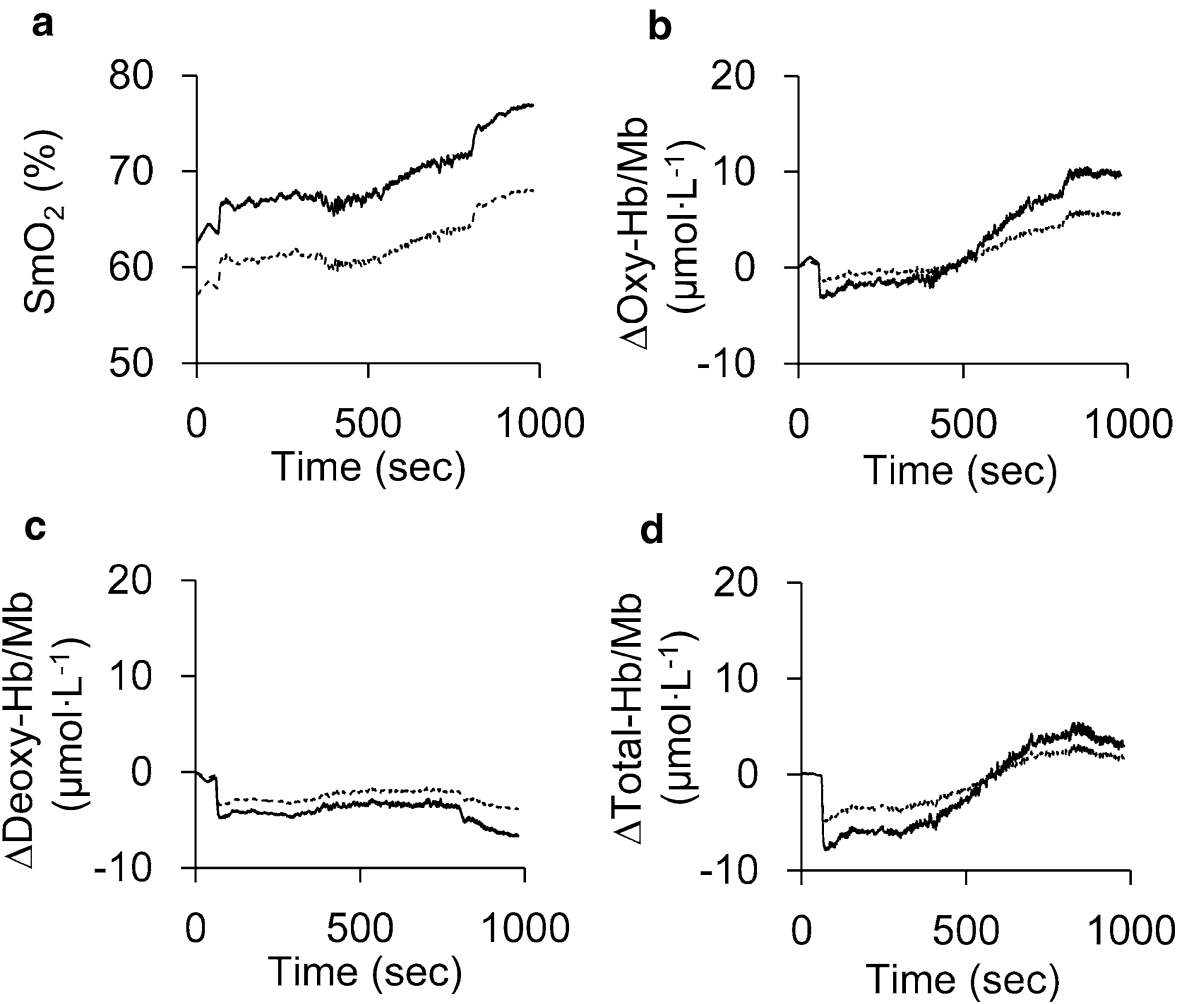
An example trace of the changes in NIRS variables in real time as the exercise proceeds. Changes in muscle oxygen saturation (SmO_2_: **a**), oxygenated hemoglobin/myoglobin (oxy-Hb/Mb: **b**), deoxygenated hemoglobin/myoglobin (deoxy-Hb/Mb: **c**), and total hemoglobin/myoglobin (total-Hb/Mb: **d**) in the vastus lateralis muscle during exercise testing expressed as a function of time (s) with (*solid lines*) and without fat correction (*dashed lines*). The figures show an example trace when fat layer thickness is 6.15 mm. Exercise testing consisted of 1 min rest, 3 min warm up, ramp exercise until exhaustion, and 3 min recovery

Figure 2 shows an example trace of the changes in NIRS variables in real time as the exercise proceeds, with and without fat correction. With fat correction, responses in NIRS variables were somewhat enhanced. Even though SmO_2_ was increased by fat correction, SmO_2_ responses were similar between with and without fat correction in this study.

**Fig. 3:**
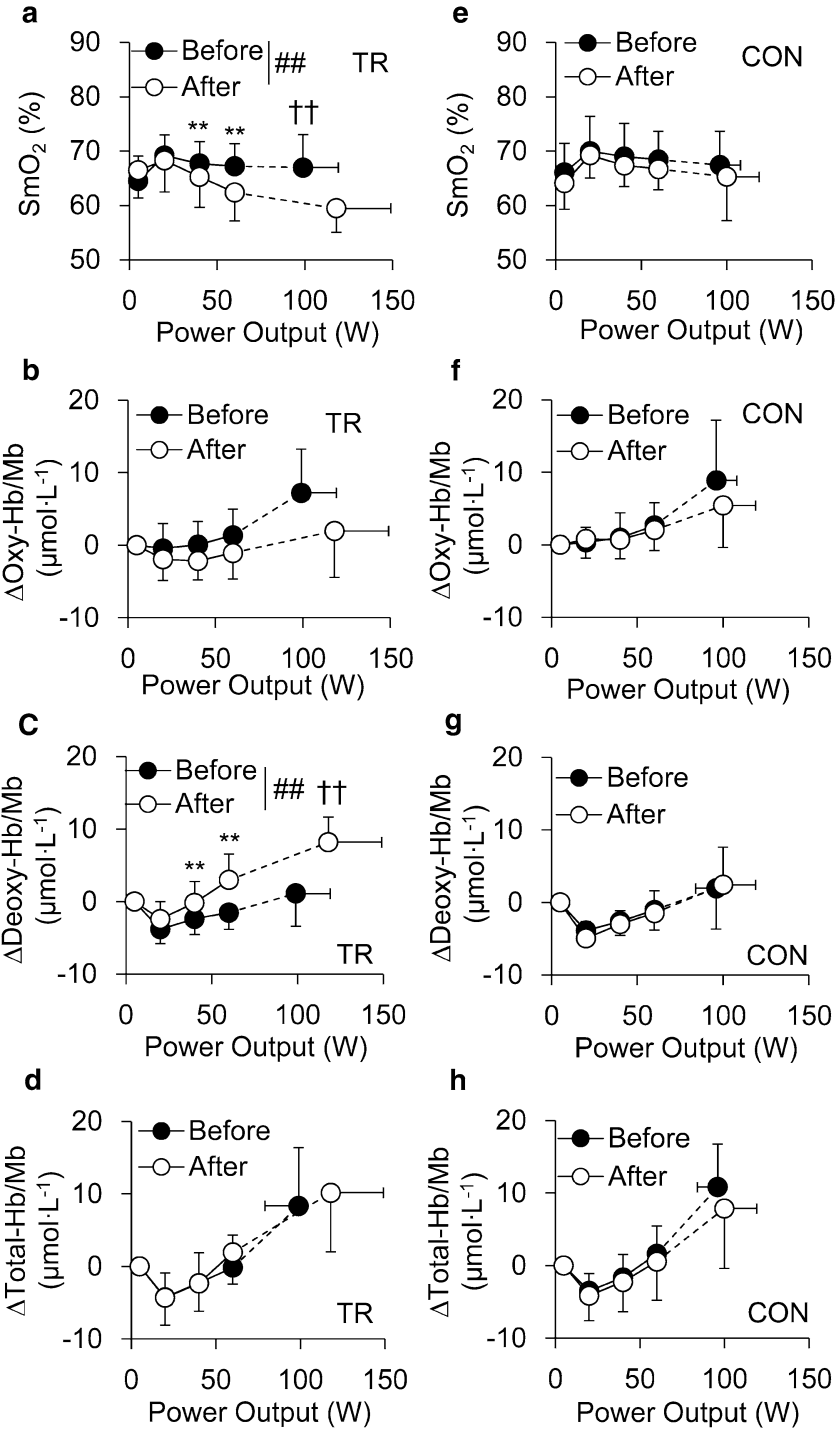
Muscle deoxygenation in TR and CON. Change in muscle oxygen saturation (SmO_2_: **a**), oxygenated hemoglobin/myoglobin (oxy-Hb/Mb: **b**), deoxygenated hemoglobin/myoglobin (deoxy-Hb/Mb: **c**), and total hemoglobin/myoglobin (total-Hb/Mb: **d**) in vastus lateralis muscle during ramp cycling exercise expressed as a function of power output intensity before (*closed circles*) and after (*open circles*) intervention in exercise training (TR) and non-training groups (CON). Significant difference between before and after intervention during submaximal exercise (*p < 0.05, **p < 0.01). Significant difference between before and after intervention at peak exercise (^†^ *p* < 0.05, ^††^ *p* < 0.01). Significant intervention (before–after) × power output interaction (^##^ *p* < 0.01). For the sake of clarity, *symbols* indicating a significant difference between power output before and after intervention have been omitted

During submaximal exercise, in TR, there were significant intervention (before–after) × power output interactions for change in SmO_2_ (*p* < 0.01, Fig. 3a) and ∆deoxy-Hb/Mb (*p* < 0.01, Fig. 3c), but not in ∆oxy-Hb/Mb (*p* = 0.21, Fig. 3b) or ∆total-Hb/Mb (*p* = 0.32, Fig. 3d). Moreover, no significant main effect for training was observed in ∆oxy-Hb/Mb (*p* = 0.18) or ∆total-Hb/Mb (*p* = 0.54) in TR. After training, SmO_2_ was significantly decreased at 40–60 W (*p* < 0.01) compared to before training. Similarly, ∆deoxy-Hb/Mb was significantly increased after training compared to before at 40–60 W (*p* < 0.01). We also found significant interactions for changes in SmO_2_ (*p* < 0.01) and ∆deoxy-Hb/Mb (*p* < 0.01) in TR when NIRS data was expressed as a function of peak VO_2_, and significantly lower SmO_2_ and higher deoxy-Hb/Mb were obtained during submaximal (40–80 % of peak VO_2_) exercise after intervention in TR. There were no significant interactions or main effects of intervention in ∆oxy-Hb/Mb and ∆total-Hb/Mb as a function of peak VO_2_. In CON, both expressed as a function of power output and a function of peak VO_2_, there were no significant changes after 12 weeks in any NIRS variables. At baseline, no significant differences were found in these variables between TR and CON during submaximal exercise.

At peak exercise, there were significant group × intervention (before–after) interactions for SmO_2_ (*p* < 0.05) and ∆deoxy-Hb/Mb (*p* < 0.05), while no significant interactions or main effect were observed in ∆oxy-Hb/Mb and ∆total-Hb/Mb. SmO_2_ at peak exercise was significantly decreased after training than before training in TR (*p* < 0.01, Fig. 3a), and consequently, lower SmO_2_ was found in TR than CON after training (*p* < 0.05). Similarly, ∆deoxy-Hb/Mb at peak exercise was significantly higher after training than before in TR (*p* < 0.01, Fig. 3c) and after in CON (*p* < 0.05).

A significant group × intervention (before–after) interaction for ∆SmO_2_ (SmO_2_ at peak exercise minus SmO_2_ at rest) was found. ∆SmO_2_ was significantly decreased after training in TR (before: 2.4 ± 5.7 %, after: −7.0 ± 3.4 %, *p* < 0.01) and after intervention in CON (TR: −7.0 ± 3.4 %, CON: 1.1 ± 3.6 %, *p* < 0.01). There was no significant interaction or main effect in T1/2reoxy (Table 2).

When NIRS variables are expressed without fat correction, similar changes were obtained in all variables. In TR, there were significant interactions for changes in SmO_2_ (*p* < 0.01) and ∆deoxy-Hb/Mb (*p* < 0.01), and significantly lower SmO_2_ and higher deoxy-Hb/Mb were observed during submaximal exercise (40 and 60 W) after training. There were no significant interactions or main effects of intervention in oxy-Hb/Mb and total-Hb/Mb. In CON, no significant interactions or main effects of intervention were found in any NIRS variables without fat correction.

### Correlation analysis

**Fig. 4:**
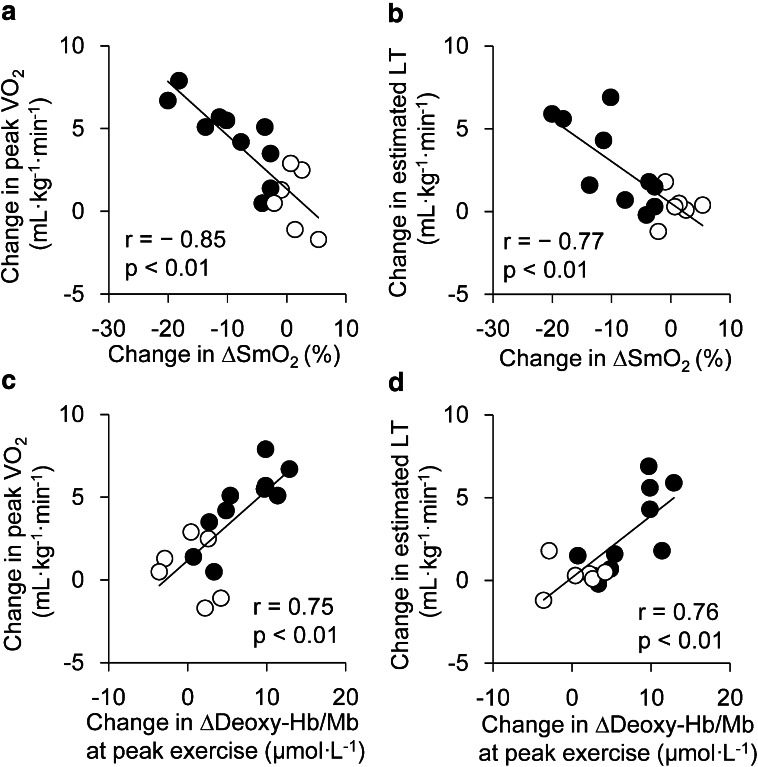
Relationship between muscle deoxygenation and aerobic capacity. Relationship between change in muscle deoxygenation by intervention and change in aerobic capacity by intervention in TR (*closed circles*) and CON (*open circles*). **a** Change in ΔSmO_2_ (ΔSmO_2_ after intervention minus ΔSmO_2_ before intervention) and change in peak VO_2_ (peak VO_2_ after intervention minus peak VO_2_ before intervention), **b** change in ΔSmO_2_ and change in estimated LT (estimated LT after intervention minus estimated LT before intervention), **c** change in Δdeoxy-Hb/Mb at peak exercise (Δdeoxy-Hb/Mb at peak exercise after intervention minus Δdeoxy-Hb/Mb at peak exercise before intervention) and change in peak VO_2_, **d** change in Δdeoxy-Hb/Mb at peak exercise and change in estimated LT. *ΔSmO*_*2*_ SmO_2_ at peak exercise minus SmO_2_ at rest, *VO*_*2*_ oxygen uptake, *LT* lactate threshold

In all subjects (TR and CON), significant negative relationships were observed between change in ∆SmO_2_ by training and increase in peak VO_2_ (*r* = − 0.85, *p* < 0.01; Fig. 4a) and estimated LT (*r* = − 0.77, *p* < 0.01; Fig. 4b). Similarly, there were positive relationships between changes in ∆deoxy-Hb/Mb at peak exercise by training and increase in peak VO_2_ (*r* = 0.75, *p* < 0.01; Fig. 4c) or increase in estimated LT (*r* = 0.76, *p* < 0.05; Fig. 4d). In TR, change in ∆SmO_2_ by training was also significantly negatively correlated with an increase in peak VO_2_ (*r* = − 0.65, *p* < 0.05) and estimated LT (*r* = − 0.73, *p* < 0.05). Similarly, significant positive relationships were observed between changes in ∆deoxy-Hb/Mb at peak exercise by training and increase in peak VO_2_ (*r* = 0.64, *p* < 0.05) or estimated LT (*r* = 0.78, *p* < 0.01).

When the influence of light scattering on NIRS variables was not corrected, the relationship was robust. Changes in ∆SmO_2_ without fat correction were significantly negatively correlated with an increase in peak VO_2_ (*r* = − 0.62, *p* < 0.05) and estimated LT (*r* = − 0.81, *p* < 0.01). There were significant positive relationships between changes in ∆deoxy-Hb/Mb at peak exercise by training and enhancement of peak VO_2_ (*r* = 0.70, *p* < 0.01) or estimated LT (*r* = 0.83, *p* < 0.01).

Workloads of training intensity (W) at end of training (*r* = 0.57, *p* = 0.08), improvement of training intensity (*r* = 0.71, *p* < 0.05), and training frequency (*r* = 0.71, *p* < 0.05) were related to the increase in peak VO_2_ by training.

## Discussion

### Muscle deoxygenation

After exercise training for 12 weeks in early post-MI patients, skeletal muscle deoxygenation during submaximal and peak exercise was enhanced, while no significant changes were observed in any variables in CON. Because deoxygenation also differed significantly at a given absolute power output, enhancement of deoxygenation is not solely attributed to the increase in maximal power output by training. Aerobic exercise training is confirmed to enhance deoxygenation measured by NIRS technique in healthy subjects (evaluated without a non-training group) (Kime et al. 2010; Prieur and Mucci 2013) and CHF patients (evaluated with a non-training group) (Mezzani et al. 2013). One possible explanation for the enhancement of muscle deoxygenation is the enhancement of muscle O_2_ consumption by aerobic training. Aerobic training increased oxidative enzyme activity in skeletal muscle of MI rats (Moreira et al. 2013). Belardinelli et al. (1995) reported that aerobic cycling training increased mitochondrial density at VL without improvement of cardiac output in CHF patients. In the present study, SmO_2_ was significantly lower and deoxy-Hb/Mb was significantly higher after training compared to before training, while total-Hb/Mb, which is an indicator of blood volume, was not significantly changed during exercise. Moreover, oxy-Hb/Mb values were decreased after training, even though changes in oxy-Hb/Mb did not reach statistical significance. Although NIRS technique measured the muscle O_2_ balance between supply and utilization, muscle deoxygenation after training in MI may be partly explained by the increase in muscle O_2_ consumption of exercising muscle. Another possibility for enhanced muscle deoxygenation during submaximal exercise is reduced muscle blood flow caused by exercise training, as seen in previous studies of healthy subjects (Saito et al. 1980) and post-MI patients (Clausen and Trap-Jensen 1970). However, VO_2_ during submaximal exercise was significantly higher after training in this study (as described below). Due to the generally positive relationship between muscle blood flow and VO_2_, we suspect that a reduction in muscle blood flow results in higher VO_2_. Taken together, we presume that the enhancement of muscle deoxygenation can be most likely be explained by muscle O_2_ utilization change, rather than flow change, during submaximal exercise.

T1/2reoxy is an index of muscle oxidative capacity, and it also reflects the balance between muscle O_2_ supply and utilization. In this study, no significant changes were observed both in TR and CON. In contrast, in the TR group, peak VO_2_ was significantly increased after intervention. These results suggest that muscle VO_2_ after peak exercise was higher after intervention compared with before in TR, and lead us to speculate that muscle blood flow at peak exercise is also increased by training. However, we should measure muscle blood flow directly in future studies.

### Muscle deoxygenation and aerobic capacity

In early post-MI patients, the effects of aerobic training on peripheral factors and its relation to peak VO_2_ have not been established. Previous studies partly demonstrated that an aerobic training intervention affected a cross-sectional area of thigh muscle (Murabayashi et al. 1997) and muscle oxidative capacity by nuclear magnetic resonance spectroscopy (Cottin et al. 1996), and the changes were related to peak VO_2_ in early post-MI patients. However, these studies did not have a non-training control group. In the TR group of the present study, changes in ∆SmO_2_ and ∆deoxy-Hb were significantly correlated with changes in peak VO_2_ and estimated LT, while no significant difference was observed in either cardiovascular or muscle deoxygenation variables in CON. An increase in aerobic capacity reduces cardiovascular-associated morbidity and mortality (Merz et al. 2009). Therefore, our findings partly support the idea that exercise training may be important for early post-MI patients to improve peripheral functions and aerobic capacity.

VO_2_ is the product of cardiac output (convective O_2_ supply) and O_2_ extraction, and in a strict sense, muscle O_2_ extraction can be estimated by deoxy-Hb/Mb or SmO_2_ when total-Hb/Mb is stable (Ferrari et al. 1997). In this study, total-Hb/Mb increased as power output increased, however, total-Hb/Mb was not different between before and after 12 weeks of intervention. Therefore, the change in deoxy-Hb/Mb and SmO_2_ between before and after training may be partly explained by increasing muscle O_2_ extraction. Okita et al. (2013) suggested that skeletal muscle dysfunction, rather than cardiac dysfunction, limits exercise tolerance in most patients with CHF. Hence, the strong relationship between change in muscle deoxygenation and increase in peak aerobic capacity in the present study may be attributed to the idea that increasing peak aerobic capacity was mainly caused by increasing muscle O_2_ extraction, rather than cardiac output, during training in early post-MI patients.

### Cardiorespiratory variables

#### VO_2_ responses

The longer MRT may be caused by low muscle O_2_ supply or muscle oxidative capacity (Meyer et al. 1998), and partly results in lower VO_2_ at a given absolute power output (Takagi et al. 2014). In this study, although the longer MRT at baseline was similar to heart disease patients in previous studies (Meyer et al. 1998; Takagi et al. 2014), the 12 weeks of training decreased MRT and increased VO_2_. In fact, previous results demonstrated that, after 6 months of exercise training, leg VO_2_ at a given absolute WR was increased in CHF patients (Hambrecht et al. 1995). Higher VO_2_ after training can be interpreted to mean that cycling efficiency was reduced and/or the contribution of oxidative metabolism was increased (i.e. reduction in contribution of phosphocreatine breakdown and/or glycolysis) (Meyer et al. 1998; Poole et al. 2012; Rossiter 2011; Takagi et al. 2014). However, it seems that the lower VO_2_ before training should not be assumed to be related to higher cycling efficiency in heart disease patients (Poole et al. 2012; Rossiter 2011). Taken together, from the results of the previous studies, aerobic training may increase muscle O_2_ supply and/or muscle oxidative capacity and, consequently, increased contribution of oxidative metabolism in early post-MI patients in this study. However, future studies are needed with a greater number of subjects.

#### HR and O_2_ pulse responses

Lower submaximal HR after TR in this study was a common adaptation of TR, as well as in previous reports of early post-MI patients (Dressendorfer et al. 1995). O_2_ pulse was increased by exercise training, as seen in a previous study of early post-MI patients (Giallauria et al. 2012). Though O_2_ pulse is the product of stroke volume multiplied by arteriovenous O_2_ difference, increased O_2_ pulse with a reduction in HR may lead us to speculate that stroke volume would possibly be increased by training.

### Influence of intensity and frequency of training

In the present study, training intensity in last session and training frequency were positively related to improvement of peak aerobic capacity. Moreover, peak VO_2_ was certainly improved by aerobic training in TR, even though both the training intensity and the frequency are low. These results indicated that both training intensity and frequency are important factors in improving peak aerobic capacity in early post-MI. Recent previous studies demonstrated that high-intensity interval training may be effective to improve aerobic capacity in coronary heart disease patients (Rognmo et al. 2012), and the risk may be the same compared with moderate intensity aerobic training in heart failure patients (Wisløff et al. 2007). However, Blumenthal et al. (1988) pointed out that “many patients experience subjective discomfort during exercise at high intensity and are therefore dissuaded from pursuing further training, despite its potential benefits”. Moreover, to our knowledge, a risk of high-intensity interval training is unknown in early post-MI patients. Therefore, we presume that frequent aerobic training seems to be one of the best options to improve aerobic capacity in early post-MI patients for now, even if the benefits are not ideal.

### Study limitations

We recognize the absence of direct measurement, such as muscle blood flow, cardiac output during exercise, or muscle biopsy, to support our findings. Secondarily, the number of subjects is quite low, especially women. Our findings should be confirmed in a larger group of subjects. Moreover, the subjects in this study were not divided randomly into TR or CON. Thus, some sampling bias cannot be ruled out. In addition, our subjects included patients treated with medications. However, our findings cannot be solely attributed to these medications, because the dosages remained stable throughout the study. Furthermore, there are some general limitations of NIRS technique (Hamaoka et al. 2007). NIRS data reflects tissue oxygenation in arterioles, capillaries, and venules of localized exercising muscle, especially in the superficial region of the muscle. The deoxygenation measured by NIRS was heterogeneous between muscles and also within a single muscle (Takagi et al. 2013). Hence, the muscle deoxygenation in early post-MI patients may be restricted to the superficial region of the distal site of the VL muscle. Because muscle recruitment affects the changes in NIRS variables during ramp cycling exercise (Chin et al. 2011), it may be that central attenuation or inhibition of muscle activity in the superficial region of the distal site of the VL muscle is responsible for the changes observed in NIRS dynamics. In particular, post-MI patients in this study had substantially preserved LVEF even before training, and the effect of training on deoxygenation may be influenced by the previously preserved LVEF. Furthermore, in early post-MI patients, aerobic capacity may be increased by very light intensity exercise such as physical activity (Dressendorfer et al. 1995). Though none of the subjects in CON were involved in training over the 12 weeks, we did not regulate their usual physical activity. Future studies are needed which include measurement of physical activity.

## Conclusions

Aerobic exercise training enhanced skeletal muscle deoxygenation during exercise in early post-MI patients. Moreover, the enhancement of muscle deoxygenation by aerobic exercise training may be related to an increase in aerobic capacity in early post-MI patients.

## Abbreviations

ANOVA: Analysis of variance
CHF: Chronic heart failure
CON: Non-training group
Deoxy-Hb/Mb: Deoxygenated hemoglobin/myoglobin concentration
HR: Heart rate
LT: Lactate threshold
LVEF: Left ventricular ejection fraction
MI: Myocardial infarction
MRT: Mean response time
NIRS: Near-infrared spectroscopy
NIR_SRS_: Near-infrared spatial resolved spectroscopy
O_2_: Oxygen
Oxy-Hb/Mb: Oxygenated hemoglobin/myoglobin concentration
RER: Respiratory exchange ratio
SmO_2_: Muscle oxygen saturation
Total-Hb/Mb: Total hemoglobin/myoglobin concentration
TR: Training group
VCO_2_: Carbon dioxide production
VL: Vastus lateralis
VO_2_: Oxygen uptake
WR: Work rate
